# Recombinant Zoster Vaccination and Risk of Postherpetic Neuralgia or Zoster Ophthalmicus

**DOI:** 10.1001/jamanetworkopen.2025.14615

**Published:** 2025-06-10

**Authors:** Ousseny Zerbo, Joan Bartlett, Bruce Fireman, Kristin Goddard, Jonathan Duffy, Jason Glanz, Allison L. Naleway, James G. Donahue, Tara C. Anderson, Nicola P. Klein

**Affiliations:** 1 Vaccine Study Center, Kaiser Permanente Northern California, Oakland, California; 2 US Centers for Disease Control and Prevention, Atlanta, Georgia; 3 Institute for Health Research, Kaiser Permanente Colorado, Denver, Colorado; 4 Kaiser Permanente Center for Health Research, Portland, Oregon; 5 Marshfield Clinic Research Institute, Marshfield, Wisconsin

## Abstract

This cohort study uses health plan data to investigate whether recombinant zoster vaccination is associated with a reduced risk of postherpetic neuralgia or zoster ophthalmicus.

## Introduction

Clinical trials have demonstrated that recombinant zoster vaccine (RZV) is highly effective in preventing postherpetic neuralgia (PHN) but have not evaluated efficacy against herpes zoster ophthalmicus (HZO).^1,2^ Observational studies have not extensively evaluated RZV effectiveness against PHN and HZO.^3,4,5^ We investigated whether RZV is associated with PHN and HZO risk reduction.

## Methods

This cohort study used data from 4 health plans in the Vaccine Safety Datalink from January 1, 2018, to December 31, 2022, and included adults aged 50 years or older. As this study used data already collected without direct contact with participants, each plan’s institutional review board approved the study with a waiver of informed consent. We followed the STROBE reporting guideline.

A person was considered partially vaccinated 30 or more days after the first dose until receipt of the second dose and fully vaccinated 30 or more days after the second dose. We used diagnosis codes to identify PHN and HZO among persons with incident herpes zoster.

Cox regression was used to estimate the hazard ratio, comparing the risk of PHN in vaccinated persons (partially or fully) with that in otherwise similar unvaccinated persons. We estimated vaccine effectiveness (VE) as 1 minus the hazard ratio, scaled as a percentage. For fully vaccinated persons, we estimated VE against PHN and HZO by time since vaccination and corticosteroid use before vaccination. We examined PHN risk among herpes zoster cases related to RZV status. Details are provided in the eMethods in Supplement 1. The threshold for significance was *P* < .05. The analysis was performed using SAS, version 9.4 (SAS Institute).

## Results

Among 1 996 885 persons followed up for 4 years (74.5% aged 50-69 years and 25.5% aged ≥70 years; 53.4% female and 46.6% male), 28.8% received both RZV doses, and an additional 9.3% received only 1 dose (Table). During follow-up, 45 333 persons (2.3%) had herpes zoster, including 6.4% and 6.9% who developed PHN and HZO, respectively.

**Table.** Sample Characteristics (N = 1 996 885)

Characteristic	Persons, No. (%)
Age group, y	
50-54	620 218 (31.1)
55-59	314 032 (15.7)
60-64	295 286 (14.8)
65-69	257 025 (12.9)
70-74	205 031 (10.3)
75-79	132 240 (6.6)
80-84	88 355 (4.4)
85-89	53 520 (2.7)
≥90	31 178 (1.6)
Sex	
Female	1 065 434 (53.4)
Male	931 451 (46.6)
Race and ethnicity^a^	
American Indian or Alaska Native	7496 (0.4)
Asian	274 858 (13.8)
Black	116 274 (5.8)
Hispanic (regardless of race)	292 962 (14.7)
Native Hawaiian or Pacific Islander	11 151 (0.6)
White	1 179 399 (59.1)
Multiracial or other^b^	57 510 (2.9)
Unknown	57 235 (2.9)
Recombinant zoster vaccine doses	
1	761 072 (38.1)
2	576 483 (28.8)
Herpes zoster	45 333 (2.3)
Postherpetic neuralgia	2922 (6.4)^c^
Herpes zoster ophthalmicus	3148 (6.9)^c^

^a^ Race and ethnicity were self-reported and available from the electronic medical record.

^b^ Includes persons who did not fit in the prespecified categories.

^c^ Denominator is people who had herpes zoster.

Against PHN, VE of full vaccination was 87% (95% CI, 83%-90%) and 69% (95% CI, 59%-76%) for partial vaccination. For full vaccination, VE was 91% (95% CI, 86%-94%) during the first year, 90% (95% CI, 82%-94%) during the second year, and 77% (95% CI, 66%-84%) after the second year. Vaccine effectiveness was 75% (95% CI, 54%-86%) in persons who received corticosteroids before vaccination vs 88% (95% CI, 84%-91%) in persons who had not. Among persons with herpes zoster, the risk of PHN was 47% (95% CI, 31%-59%) lower in those vaccinated vs unvaccinated. Against HZO, VE of full vaccination was 78% (95% CI, 73%-82%) and of partial vaccination, 68% (95% CI, 58%-76%). Although these confidence intervals overlap, the VE estimates differed significantly (*P* = .03). The VE for full vaccination was 80% (95% CI, 73%-85%) during the first year, 77% (95% CI, 67%-84%) during the second year, and 74% (63%-82%) after the second year (Figure).

**Figure.**
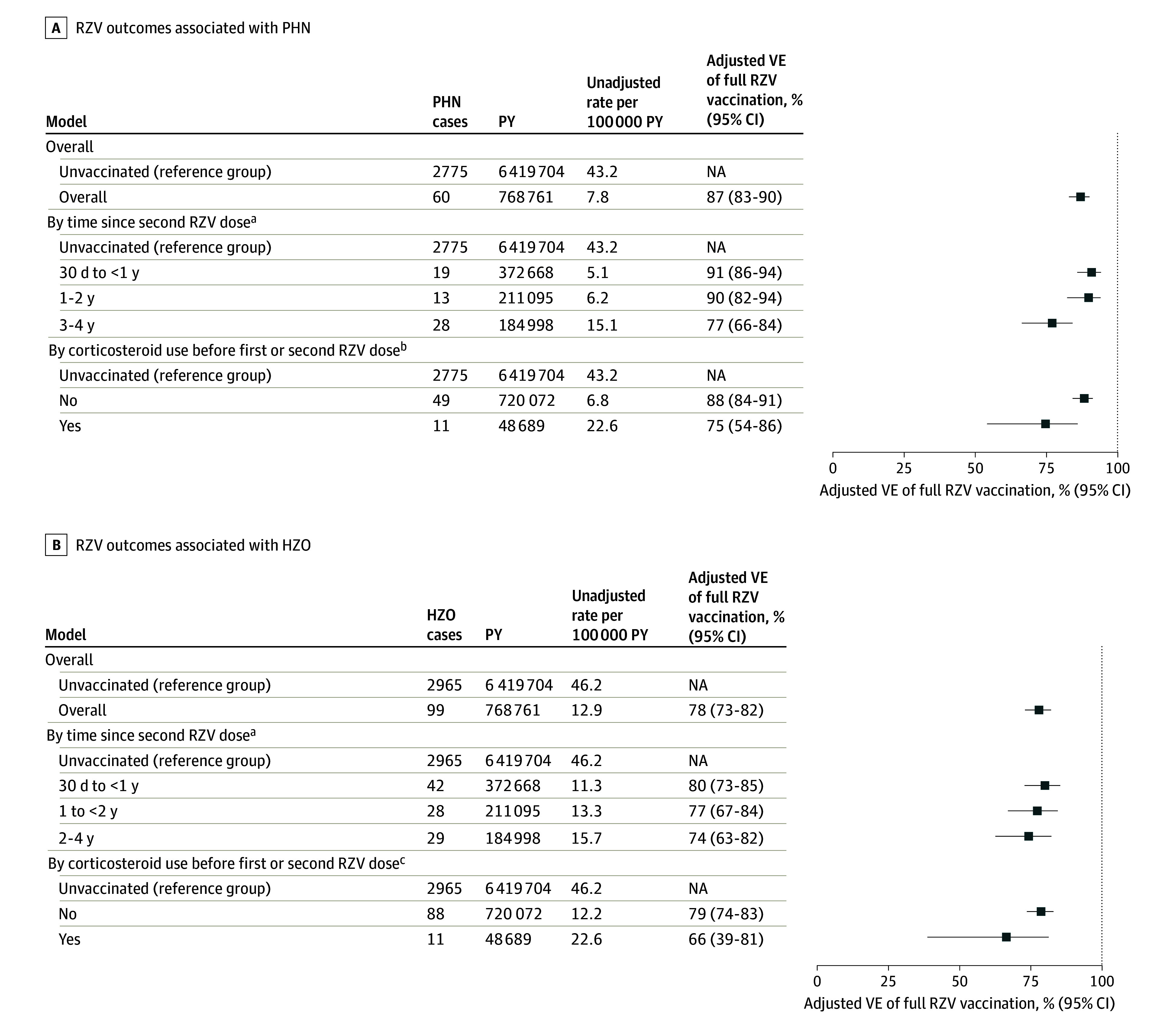
Outcomes of Full Recombinant Zoster Vaccine (RZV) Associated With Postherpetic Neuralgia (PHN) and Herpes Zoster Ophthalmicus (HZO), 2018-2022 Shown is a summary of vaccine effectiveness (VE) estimates for fully vaccinated persons obtained from 3 models: 1 model with a 5-level measure of RZV vaccination status (unvaccinated, 1-29 days after the first dose, ≥30 days after the first dose, 1-29 days after the second dose, and ≥30 days after the second dose) and 2 models similar to that model, except that fully vaccinated persons were divided into subgroups based on time since second dose and corticosteroid use before vaccination. All 3 models were conditioned on Vaccine Safety Datalink site and calendar time and adjusted for age; sex; race and ethnicity; zoster vaccine live status; corticosteroid use; influenza vaccination; hospital admission; outpatient visit frequency; and diagnoses for diabetes, chronic obstructive pulmonary disease, coronary heart disease, obesity, and hypertension. NA indicates not applicable; PY, person-year. ^a^*P* = .002 comparing VE during 30 days to less than 1 year vs during 2 to 4 years. ^b^*P* = .02 comparing VE estimates by corticosteroid use. ^c^The differences were not statistically significant.

## Discussion

In this cohort study, 2 doses of RZV was estimated to reduce the risk of PHN by 87% and of HZO by 78%. While protected by only 1 RZV dose, VE was somewhat lower for both PHN and HZO, underscoring the importance of the second dose. After the second dose, VE against PHN waned by a small amount. The trajectory of waning effectiveness over a longer time since vaccination needs further investigation.

Although corticoidsteroid users may have lower VE against PHN, RZV may have prevented more PHN due to immunocompromise and higher risk than nonusers.^6^ Our finding that RZV was associated with a reduced risk of PHN in breakthrough herpes zoster cases suggests that RZV may prevent PHN by preventing herpes zoster and reducing the risk of PHN in herpes zoster cases.

This study was limited by its case finding, which missed persons with PHN or HZO who did not seek care. Nonetheless, in this large cohort, we found RZV to be associated with reducing the risk PHN and HZO.

## Notes

Supplement 1.
eMethods.

eReferences.

Supplement 2.
Data Sharing Statement
