# Supplementary material for: Recombinant Zoster Vaccination and Risk of Postherpetic Neuralgia or Zoster Ophthalmicus
Source: JAMA Netw Open. 2025 Jun 10;8(6):e2514615. doi: 10.1001/jamanetworkopen.2025.14615 (PMC12152699; doi:10.1001/jamanetworkopen.2025.14615)
Supplement: Supplement 1. — eMethods. eReferences. [file jamanetwopen-e2514615-s001.pdf]

Supplemental Online Content

Zerbo O, Bartlett J, Fireman B, et al. Recombinant zoster vaccination and risk of postherpetic neuralgia or zoster ophthalmicus. *JAMA Netw Open*. 2025;8(6):e2514615.
doi:10.1001/jamanetworkopen.2025.14615

eMethods

eReferences

This supplemental material has been provided by the authors to give readers additional information about their work.

© 2025 Zerbo O et al. *JAMA Network Open*.

eMethods

Study Setting and population

The study setting and population have been previously described [1]. Briefly, the study was conducted at 4 sites within the Vaccine Safety Datalink, a collaboration between the Centers for Disease Control and Prevention (CDC) and integrated healthcare organizations with electronic health records[2]. The study included 3 Kaiser Permanente sites (Northern California, Colorado, and Northwest) and Marshfield Clinic (Wisconsin). Each site's institutional review board approved the study.

The study included VSD members who were age eligible for RZV vaccination and had 12 months of continuous membership before study entry. They were age 50 years or older when the study began on January 1, 2018, or reached age 50 years before December 31, 2022, when follow-up ended for incident HZ cases. We followed study participants until they were diagnosed with HZ, disenrolled from the health plan, died or until the end of the study period (December 31, 2022). All HZ cases were then followed to ascertain PHN and HZO. We excluded the few members who had already received RZV and those with an HZ diagnosis in the year before study entry.

Study outcomes

We first identified incident HZ cases requiring a diagnosis code (B02.xx International Classification of Diseases, 10th Revision [ICD-10]) and an antiviral prescription. We previously found that the predictive positive value of incident HZ case of this definition was 96.7% [1]. Among these incident HZ cases, we then identified the subset who had a PHN diagnosis (ICD-10 codes B02.22 and B02.23) between 90 days and 365 days after their initial HZ diagnosis, and the

© 2025 Zerbo O et al. *JAMA Network Open*.

subset who had a HZO diagnosis (ICD-10 codes B02.3x) within 30 days of their HZ diagnosis. For HZO cases, we required that the HZO diagnosis be given at an ophthalmology department except at one site where the diagnosis could be given anywhere.

Exposure

As previously described, the exposure was a time-varying indicator of RZV vaccination status[1]. All participants started follow-up unvaccinated, and their status subsequently changed if vaccinated. For every day during follow-up, participants were categorized as either unvaccinated, 1 to 29 days after the first dose, 30 or more days after the first dose, 1 to 29 days after the second dose, or 30 or more days after the second dose. Participants were considered "fully vaccinated" 30 or more days after they received the second dose and "partially vaccinated" 30 or more days after they received the first dose.

Covariates

We adjusted VE estimates for time-fixed covariates, including sex and race, and for time-varying covariates, including age in 5-year groups, zoster vaccine live (ZVL) vaccination status, influenza vaccination status, comorbidities, outpatient visit frequency, hospital admission, and corticosteroid use as an indicator of possible immune compromise. We measured corticosteroid use as an incomplete proxy for immunocompromise status that was feasible to measure across all study sites. All covariate specifications were described in detail elsewhere[1].

Statistical analysis

We calculated the incidence of PHN in the study population and the percentage of HZ cases who developed PHN by RZV vaccination status and age group.

© 2025 Zerbo O et al. *JAMA Network Open*.

We used Cox regression to fit several models comparing the risk of PHN in persons who were vaccinated with the risk in otherwise similar persons who were unvaccinated. Each model was specified with a calendar timeline and stratified by site. Each model included the time-fixed and time-varying covariates listed above. Thus, vaccinated persons were compared with unvaccinated persons in risk sets that comprised persons at risk for PHN on the same day at the same site. The first model included a 5-level measure of RZV vaccination status: unvaccinated, 1 to 29 days after the first dose, 30 or more days after the first dose, 1 to 29 days after the second dose, and 30 or more days after the second dose. Unvaccinated persons were the reference group. We estimated the PHN hazard ratio for persons who were fully vaccinated and those who were partially vaccinated. We estimated VE as 1 minus the hazard ratio, scaled as a percentage.

We next fit two additional Cox models to examine whether the effectiveness of RZV against PHN in fully vaccinated persons varied by time since second dose and by corticosteroid use before either dose. To examine VE by time since the second dose, we subdivided follow-up after the second dose into three intervals (30 days to <1 year, 1 to <2 years, or 2 to 4 years). To examine VE by corticosteroid use, we subdivided fully vaccinated persons based on whether they used oral or intravenous corticosteroids during the 90 days before either dose.

To examine VE against HZO, we used the same models that were used to examine VE against PHN.

Finally, in a secondary analysis, we also examined risk of PHN among HZ cases in relation to RZV status.

© 2025 Zerbo O et al. *JAMA Network Open*.

eReferences

1. Zerbo O, Bartlett J, Fireman B, et al. Effectiveness of Recombinant Zoster Vaccine Against Herpes Zoster in a Real-World Setting. *Ann Intern Med*. Feb 2024;177(2):189-195. doi:10.7326/M23-2023
2. McNeil MM, Gee J, Weintraub ES, et al. The Vaccine Safety Datalink: successes and challenges monitoring vaccine safety. *Vaccine*. Sep 22 2014;32(42):5390-8. doi:10.1016/j.vaccine.2014.07.073

© 2025 Zerbo O et al. *JAMA Network Open*.
